# Biomarkers of Innate Immunity and Immunological Susceptibility to Viral Infection in Patients with Alcoholic Cirrhosis

**DOI:** 10.3390/biomedicines12020336

**Published:** 2024-02-01

**Authors:** Isabel Legaz, Elena Navarro-Noguera, Aurelia Collados-Ros, Jose Miguel Bolarín, Manuel Muro

**Affiliations:** 1 Department of Legal and Forensic Medicine, Biomedical Research Institute of Murcia (IMIB), Regional Campus of International Excellence “Campus Mare Nostrum”, Faculty of Medicine, University of Murcia (UMU), 30100 Murcia, Spain; 2 Digestive Medicine Service, Hospital Clinico Universitario Virgen de la Arrixaca (HCUVA), 30120 Murcia, Spain; 3 Immunology Service, University Clinical Hospital “Virgen de la Arrixaca”—IMIB, 30120 Murcia, Spain

**Keywords:** alcohol, cirrhosis, human toxicology, KIR/HLA genes, NK cells, virus

## Abstract

Background: The harmful effect of alcohol on the immune system may be due to both a direct action of the alcohol or its metabolites on immune cells as an indirect action modifying the different mechanisms of intercellular interaction. The interplay between stimulatory (aKIR) and inhibitory (iKIR) natural killer (NK) cell receptors and their corresponding human leukocyte antigen (HLA) ligands influences the outcome of virus infection. The aim was to analyze the influence of the KIR/HLA pair genetic profile in male alcoholic cirrhosis (AC) patients with and without viral infections to find susceptibility biomarkers that can help establish the risks and prevent viral infections. Methods: A total of 281 male AC patients were analyzed. The sociodemographic characteristics, viral hepatitis C (HCV), hepatitis B (HBV), and cytomegalovirus (CMV) infections were analyzed. Genomic DNA was extracted, and genetic the *KIR*/*HLA* profiles were investigated. A total of 6 *KIR* genes and their corresponding ligands (HLA-C) were analyzed. Patients were grouped into two groups: with and without associated viral infection. Results: A statistically significant increase in the combination of *KIR2DL2+*/*C1C1* was observed in male AC patients with viral infection compared to those without viral infection (45.9% vs. 24.5%, *p* = 0.021). The analysis of *KIR2DL3+*/*C1+* showed a high frequency comparing healthy controls and male AC patients without virus infection (85% vs. 76.4%; *p =* 0.026). The analysis of *KIR2DL3+*/*C2C2* frequency showed a statistically significant increase comparing male AC patients without viral infection and healthy controls (23.6% vs. 15%; *p =* 0.026). Conclusions: The genetic *KIR2DL2+*/*C2C2* profiles may play a significant role in determining the vulnerability of male AC patients to viral infections, providing valuable insights for future research and potential therapeutic interventions.

## 1. Introduction

The harmful effect of alcohol on the immune system may be due to both a direct action of the alcohol or its metabolites on immune cells as an indirect action modifying the different mechanisms of intercellular interaction [1,2,3]. Neither mechanism excludes the other, and possibly both coexist in the immunological injury associated with alcohol consumption [4,5]. The optimal functioning of the immune system requires a high degree of interaction, modulation, and regulation between the different cell types that it comprises [6]. According to research conducted in vitro, acute ethanol consumption in humans has been shown to either increase or reduce the activity of natural killer (NK) cells [7], depending on when the measurement is taken [8,9,10]. On the other hand, the activity of NK cells obtained from chronic alcoholics can be normal, although there is associated liver disease [7,8,9]. Without chronic ethanolic liver disease, NK cell activity tends to be decreased or even suppressed [10].

In patients with HCV infection, alcohol intake hastens liver damage and reduces interferon alfa’s (IFN-α) anti-hepatitis C virus (HCV) activity [11]. Alcohol’s potential to accelerate HCV disease progression and improve HCV replication is uncertain [12,13,14]. The capacity to study the relationship between alcohol and HCV replicon expression has been made possible by the availability of hepatic cells that contain the HCV replicon [11].

IFN-based regimens, a poorly tolerated medication, were formerly used to treat chronic HCV infection [15,16]. Patients who actively used alcohol in the previous year were more likely to discontinue IFN-based HCV treatment during the IFN period; therefore, many medical professionals were reluctant to treat them [17]. Classically, the existence of pre-transplant viral infection has been investigated in HBV, HCV, and CMV infections in relative studies in liver and bone marrow transplantation and also in liver pathology as previously published [18] where the influence of these factors in rejection and outcome is established.

On the other hand, NK cells are part of the innate immune system and play a significant role in defense against viral infections and tumor cells [19,20,21]. NK cells express surface-binding proteins (killer cell immunoglobulin-like receptors or KIRs) that bind ligands on most cells’ surfaces. The interplay between stimulatory and inhibitory NK cell receptors and their corresponding human leukocyte antigen (HLA) ligands influences the outcome of acute HCV infection and other infections [22,23]. Previous studies demonstrated that *KIR* receptor genes and *HLA class I* and *II* loci helped determine the clinical outcome of HCV infection [22,23,24,25]. Infections in alcoholic cirrhosis (AC) patients significantly cause morbidity and mortality [11].

The aim was to analyze the influence of the *KIR*/*HLA* pair genetic profile in male AC patients with and without viral infections to find susceptibility biomarkers that can help establish risks and prevent viral infections.

## 2. Patients and Methods

### 2.1. Patient Enrollment

**Figure 1:**
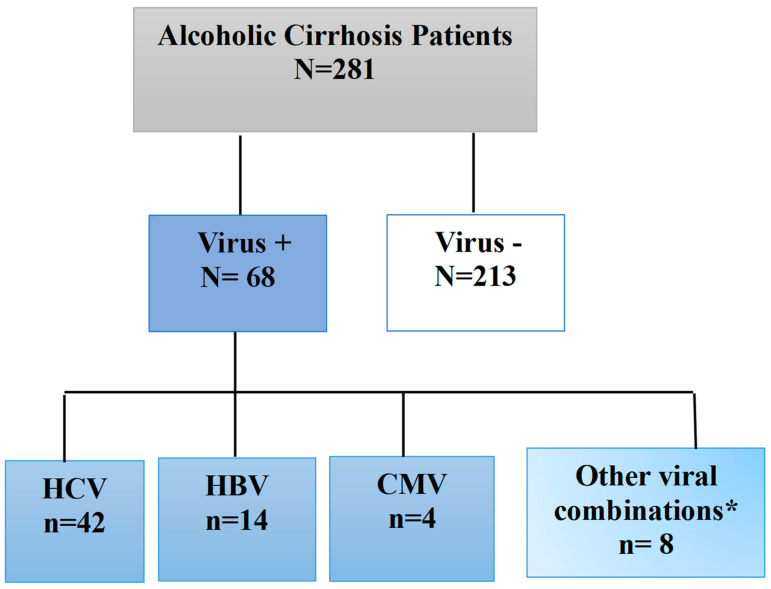
Different types of patients studied in this study are shown in the graphic. N, total number of individuals; n is the number of individuals in each subgroup; AC, alcoholic cirrhosis. CMV: Cytomegalovirus. HBV: Hepatitis B virus. HCV: Hepatitis C virus; Other viral combinations* included HCV + HBV, HCV + CMV, HBV + CMV.

A retrospective analysis was done on the medical records of 281 male AC patients with and without virus infection at the University Clinic Hospital Virgen de la Arrixaca in Spain (Figure 1). The exclusion criteria were the following: children’s or women’s AC patients were removed due to the scarce sample size. Age-related sociodemographic information, HCV, hepatitis B (HBV), cytomegalovirus (CMV) infections, and the genetic *KIR*/*HLA* profile were investigated.

The Model for End-Stage Liver Disease score in male AC patients was 14.23 ± 0.37 (mean ± SEM). In this study, the mean age was comparable across all patients studied. Men who had a history of drinking alcohol, and had cirrhosis were required to meet the inclusion criteria. In addition, 319 age-matched male individuals were used as a healthy control cohort compared to the overall male AC population. In each case, the median scores for the two populations were similar. Before participating in the trial, each patient provided informed consent for inclusion. The HUVA Ethics Committee (PI19/01194) approved the study, which was carried out per the Declaration of Helsinki.

### 2.2. Alcohol Cirrhosis Diagnostic Criteria

Clinical, radiographic, and biochemical indicators were used to diagnose AC [26]. In cases where the self-report of alcohol intake was negative, the relatives’ opinions were considered. Cirrhosis was discovered through a routine scan, ultrasound, or clinical examination, because the disease usually has no symptoms in its early stages. Other times, the illness went undiagnosed until the second stage of decompensated cirrhosis, at which point problems such as ascites, upper gastrointestinal hemorrhage, and encephalopathy manifested. As previously reported [27], cases of suspected cirrhosis were confirmed using analysis and imaging methods.

### 2.3. Viral Infection Diagnosis

Viral infections such as HCV, HBV, and CMV were analyzed. A qualitative immunoassay (AxSYM HCV v 3.0, Abbott, Wiesbaden-Delkenheim, Germany) was used to identify anti-HCV antibodies and ascertain the presence of HCV. If the test was positive for anti-HCV antibodies, an RNA test for HCV was performed to confirm chronic infection The manufacturer’s instructions verified the results using immunoblotting technology (RIBA), real-time (RT)-PCR (REAL, C.E. Durviz, Valencia, Spain) or Roche COBAS^®^ Ampliprep TNAI/TaqMan^®^ 48 RUO Assay, Thermofisher Scientific, Hempstead, UK.

HBV infection was detected using a radioimmunological approach to measure the HBV surface antigen (HBsAg), and CMV infection was detected using an immunoassay (Liason^®^ CMV-IgG, DiaSorin, Saluggia, Italy) to test for anti-CMV IgG antibodies. The first diagnosis of the CMV infection was made using IgG antibodies at a concentration of 0.6 IU/mL. An RT-PCR was utilized to confirm the results of these positive samples (LightCycler^®^ CMV-Quant-kit, Roche, Indianapolis, IN, USA). Although it could be questionable to use CMV IgG as the screening criteria for CMV diagnosis due to CMV IgG would need 15 days to turn positive after exposure, we reviewed all cases in our study and no missed cases were found.

### 2.4. KIR and HLA Typing

The QIAamp DNA Blood Mini Kit (QIAGEN, Hilden, Germany) was used to extract genomic DNA from peripheral blood, as advised by the manufacturer and previously reported [28], and quantified by NanoDrop (Thermo Scientific™, Boston, MA, USA).

KIR genotyping was conducted in patients and healthy individuals utilizing Luminex^®^ technology and sequence-specific oligonucleotides (PCR-SSO) (Tepnel Lifecodes, Stanford, CT, USA). This technique reveals inhibitory *KIR2DL1-3* and *KIR3DL1-3* genes and activates *KIR2DS1-S5*, *KIR3DS1*, and *KIR2DL4*. This approach failed to separate the genes *KIR2DL5A* and *KIR2DL5B*; however, it did identify *KIR2DP1* and *KIR3DP1* pseudogenes. Both genes were investigated, since *KIR2DL2* is frequently related to *KIR2DS2* and their individual effects could not be differentiated in our population. Patients were divided into two groups for this study according to the presence of inhibitory *KIR* genes (iKIRs) and activating *KIR* genes (aKIRs). Constitutive *KIR* genes and pseudogenes were excluded in this study.

The Dynal RELI^TM^ SSO HLA-C Kit (Dynal Biotech ASA, Oslo, Norway) was used for genotyping *HLA-I* at a degree of resolution required to identify the dimorphism at position 80 on the 1-helix. The two main *HLA-C KIR* ligand allele groups, *HLA-C1* and *HLA-C2*, as well as the three separate HLA-C genotypes, *C1C1*, *C1C2*, and *C2C2*, were determined based on this dimorphism [29,30,31].

### 2.5. Statistical Analysis

Demographic and outcome data were entered into a database (Microsoft Access 2.0; Microsoft Corporation, Seattle, WA, USA) and analyzed with SPSS v27.0 (SPSS software Inc., Chicago, IL, USA). All data were expressed as the mean, standard deviation, or percentage. Pearson’s chi-square and two-tailed Fisher’s exact tests were employed to compare classified variables between groups, while a two-sided Student *t*-test and the nonparametric Mann–Whitney test were used to compare mean values. The statistical significance level was set at *p* = 0.05. The odds ratio (OR) and 95% confidence interval (CI) were determined to evaluate relative risk.

## 3. Results

### 3.1. Clinical and Sociodemographic Characteristics

The sociodemographic and main clinical characteristics of the total cohort (n = 281) of male AC patients are shown in Table 1. This study excluded pediatric and adult patients who could not have specified testing performed. The mean age was similar in all the analyzed male AC patients with and without the associated virus. There were no statistically significant differences between them. In our cohort of male AC patients, 68 patients (24.2%) with associated viral infections were observed.

Table 1 shows the frequency of each virus type in male AC patients analyzed in this study. HCV infection was the most representative (61.8%), which added to the percentage of coinfection with HBV (5.9%) and CMV (2.9%), representing 70.6% of patients infected by HCV. HBV infection was represented in 20.6%. In addition, HBV occurs in a tiny percentage associated with CMV (2.9%, n = 2). CMV infection represented 5.9% of AC patients.

### 3.2. Analysis of KIR Genes and Their Corresponding HLA-C Ligands in AC Patients with and without Virus Infections

The frequency of each *KIR* gene (*iKIRs* and *aKIRs*) with their corresponding HLA-I ligands (C1, C2) in male AC patients with and without virus infection was analyzed (Table 2).

The analysis of *KIR2DL1+*/*S1−* with and without its epitope (*C2+*/*C2−*) showed a similar frequency distribution in the healthy population compared with total AC patients (*p* = 0.817). By contrast, the analysis of *KIR2DL3+* with C1+ showed a higher frequency in the control group versus total AC patients (85% vs. 75.9%, respectively); this decrease in AC patients was statistically significant. (OR = 1.801; 95% CI: 1.155–2.809, *p =* 0.010). Similar results were also obtained when comparing healthy controls and male AC patients without virus infection (85% vs. 76.4%; OR = 1.758; 95% IC: 1.092–2.831, *p =* 0.026).

Regarding the activating *KIRs* (*aKIRs*), the frequency of *KIR2DS1+* was also analyzed with the epitope (*C2+*/*C2−*), and no statistically significant differences were also found between the control group and male AC patients (*p =* 0.670). Next, the presence of *KIR2DS4+* with its two known ligands (C1+ and C2+) was analyzed. It was observed that the frequency of *KIR2DS4+*/*C1+* AC patients was significantly lower in the total male AC patients compared to controls (76.3% vs. 84.7% OR = 1.719; 95% CI: 1.123–2.630, *p* = 0.013). Similar results were also observed when comparing total male AC patients without virus infection (76.3%) and control individuals (84.7%; OR = 1.714; 95% CI: 1.083–2.711, *p =* 0.024).

Furthermore, the combination of *KIR2DS5* with the C1+ ligand has also been less frequent in the total group of male AC patients compared to the control group (84.3% vs. 72.9%), although in this case without reaching statistical significance (*p =* 0.072). On the contrary, the combination of the C2+ ligand with *KIR2DS5* appeared to increase in the group of total male AC patients compared to the control group (74% vs. 67.5%), but the difference observed was not statistically significant (*p =* 0.410).

### 3.3. Analysis of KIR Genes and Their Genotypes HLA-C in Male AC Patients with and without Virus Infections

The combination of *KIR* genes with HLA-C genotypes was also analyzed in male AC patients with and without viral infections and healthy controls (Table 3).

The analysis showed how the *KIR2DL1+*/*S1*− with *C2C2* genotype (14.9%) was diminished in healthy controls versus total AC patients (21.2%). However, the observed differences did not reach statistical significance (*p =* 0.159). On the other hand, no significant changes were observed for these combinations when AC patients with and without viral infection were compared (17.8% vs. 22.5%; *p =* 0.666).

**Figure 2:**
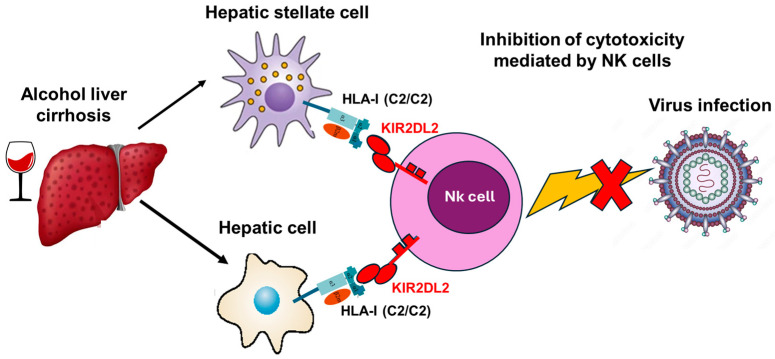
Hypothetical scheme of the inhibition of cytotoxicity mediated by NK cells in AC patients with the genetic profile (*KIR2DL2*/*C2C2*) and high susceptibility to associated viral infection. It is shown that the presence of *KIR2DL2*/*C2C2* in the patient produces cellular inhibition of the NK cell, which is incapable of carrying out its cytotoxic activity to eliminate viral infections.

Then, the combination of *KIR2DL2* with the different *HLA-C* genotypes was also analyzed, and a statistically significant increase in the combination of *KIR2DL2+*/*C1C1* was observed in AC patients with viral infection (Figure 2) compared with these patients without viral infection (45.9% vs. 24.5%, OR = 0.382; 95% CI: 0.175–0.837, *p* = 0.021).

The analysis of *KIR2DL3+* and *HLA-C* genotypes showed an increase in the frequency of the *C2C2* genotype in total AC patients (24.1%) compared to the control population (15%), this difference being statistically significant (OR = 0.555; 95% CI: 0.356–0.866, *p =* 0.010). Similar results were observed when comparing AC patients without viral infection and healthy controls (OR = 0.569; 95% CI: 0.353–0.916, *p =* 0.026).

The frequency of *KIR2DS4+*/*C2C2* in total AC patients (23.7%) was lower than in healthy controls (23.7% vs. 15.3%; OR = 0.582; 95% IC: 0.380–0.891, *p* = 0.013). Similar results were observed when comparing total AC patients without virus infection (23.7%) and controls (15.3%; OR = 0.584; 95% IC: 0.369–0.923, *p =* 0.024).

## 4. Discussion

This study aimed to investigate the genetic profile of the *KIR*/*HLA* pair in male AC patients with and without viral infections to uncover susceptibility biomarkers that can assist in establishing risks and preventing viral infections in AC patients as a new approach aimed at detecting the profile which reflects greater vulnerability to viral infection and worsening liver functions.

Our results showed that the most common viral infection in AC patients was HCV, followed by HBV and CMV. The relationship between alcohol and HCV is well-established, with evidence suggesting that alcohol consumption can worsen the progression and severity of HCV infection [32,33]. The combined effects of alcohol and HCV on the liver can lead to increased liver damage, fibrosis, and even cirrhosis [31]. Moreover, alcohol use has been shown to decrease the effectiveness of HCV treatment and increase the risk of developing hepatocellular carcinoma (HCC) in HCV-infected individuals [34,35]. Therefore, healthcare professionals and individuals diagnosed with HCV must acknowledge the detrimental role of alcohol in exacerbating the disease. By abstaining from alcohol or practicing moderation, individuals can significantly improve their chances of healthier outcomes and ultimately reduce the burden of HCV [36].

On the other hand, alcohol consumption has a detrimental effect on HCV infection and the functioning of NK cells, leading to an increased risk of liver damage and disease progression [31,37,38]. Evidence consistently demonstrates that alcohol induces oxidative stress, impairs immune response, and promotes HCV replication within the liver. Furthermore, alcohol interferes with the role of NK cells in recognizing and eliminating HCV-infected cells, diminishing their antiviral function [39,40,41]. Evidence suggests that NK cells, through their membrane receptors, play dual roles in developing and progressing liver fibrosis, performing both profibrotic and antifibrotic actions [42]. On the other hand, the role of NK cells in controlling viral infections is also known [43,44].

KIR receptors on the NK cell and their specific ligands, HLA class I (MHC-1) molecules, also play an essential role in the antifibrotic effect [45]. Inhibitory KIR receptors (iKIR) present on the NK cell bind to their ligands, HLA class I (MHC-1) molecules present on the hepatic stellate cell (HSC), inhibiting its destruction. When liver damage occurs, the expression of inhibitory KIR receptors decreases; therefore, the inhibitory effect on the destruction of HSCs decreases [46]. The binding of iKIR with MHC-1 sets in motion a series of mechanisms that will favor apoptosis of stellate cells [47]. Our results showed a statistically significant increase in viral infection in male AC patients with the *KIR2DL2+* with *HLA-C1*/*C1* combination (Figure 2). This molecular interaction produces an inhibition of the cytotoxic function of NK cells that prevents the innate immune system from eliminating virus-infected cells. Similar results were obtained in a study with chronically infected patients with HIV-1, which identified two *KIR*/*HLA* combinations, *KIR2DL2*/HLA-C*12:02 and *KIR2DL2*/HLA-C*14:03, that impact suppression of HIV-1 replication [48].

The interaction of stimulatory and inhibitory *KIRs* with their corresponding HLA ligands is believed to influence the outcome of acute Hepatitis C viral (HCV) infection, leading to either chronic viremia (CV) or spontaneous viral clearance (SC). The ability of alcohol to increase HCV disease progression and improve HCV replication is unknown [12,13,14]. The availability of hepatic cells containing the HCV replicon has enabled researchers to investigate the link between alcohol and HCV replicon expression [11].

Interferon-based regimens, which are poorly tolerated, were formerly used to treat chronic hepatitis C (HCV) infection [15,16]. Patients who had used alcohol the previous year were more likely to abandon interferon-based HCV treatment during the interferon period; therefore, many doctors were hesitant to treat them [17].

Other studies [49,50] observed an association between inhibitory *KIR* genes and their specific ligands (*KIR 2DL3*/*3DL3* with *HLA-C1*/*C1*) in patients infected with HCV but in this case related to CS. On the other hand, Azocar et al. [51] described the association of DQB1*0501 with CV. Also, new genetic interactions have been described between the *KIR* gene *2DL3* and HLA-DRB*1201 in SC of HCV [27].

## 5. Conclusions

In conclusion, this study allows us to expand the knowledge between innate immunity and alcoholic cirrhosis, allowing (1) to delve into the molecular mechanisms carried out by innate immunity that can trigger a greater susceptibility to viral infections in the AC patient, and (2) to search for therapeutic strategies that can prevent and mitigate the effect of the inhibition of NK cytotoxicity in the patient, since it can also make him susceptible to the development of hepatocellular carcinoma. The screening of *KIR* genes and *HLA-C* genotypes identified that *KIR2DL2*/*C2C2* genetic profiles may play an important role in determining the vulnerability of male AC patients to viral infections, providing valuable information for future research to delve into the possibility of enhancing the activation of the cytotoxic activity of the NK cells of these patients.

## Figures and Tables

**Table 1:** Characterization of the male AC study population.

	n (%)	Mean Age (Years ± SEM)
Total male AC patients	281	53.02 ± 0.43
Without viral infections	213 (75.8)	52.47 ± 1.33
With viral infections	68 (24.2)	53.06 ± 0.45
HCV	42 (61.8)	
HBV	14 (20.6)	
CMV	4 (5.9)	
HCV + HBV	4 (5.9)	
HCV + CMV	2 (2.9)	
HBV + CMV	2 (2.9)	

n, is the number of individuals in each subgroup; AC, alcoholic cirrhosis.; CMV: Cytomegalovirus. HBV: Hepatitis B virus. HCV: Hepatitis C virus; SEM: Standard error of the mean.

**Table 2:** Analysis of *KIR* genes and their epitopes *HLA-I* ligands in male AC patients with and without virus compared with healthy controls.

				Male AC Patients					
		Healthy Controls N = 319	Total Patients N = 281		with Virus N = 68	without Virus * N = 213			
*KIR* Genes	HLA-I Ligand	n (%)	n (%)	P1	n (%)	n (%)	P2	P3	P4
*iKIRs*									
*KIR2DL1+*/*S1−*	*C2+*	133 (68.2)	109 (69.9)	0.817	30 (66.7)	79 (71.2)	0.860	0.609	0.570
	*C2–*	62 (31.8)	47 (30.1)		15 (33.3)	32 (28.8)			
*KIR2DL2+*	*C1+*	166 (83.4)	109 (76.2)	0.128	31 (83.8)	78 (73.6)	1.000	0.050	0.265
	*C1–*	33 (16.6)	34 (23.8)		6 (16.2)	28 (26.4)			
*KIR2DL3+*	*C1+*	233 (85)	183 (75.9)	0.010 ^a^	44 (74.6)	139 (76.4)	0.057	0.026 ^c^	0.861
	*C1–*	41 (15)	58 (24.1)		15 (25.4)	43 (23.6)			
*aKIRs*									
*KIR2DS1+*	*C2+*	78 (67.2)	81 (70.4)	0.670	15 (65.2)	66 (71.7)	1.000	0.546	0.611
	*C2–*	38 (32.8)	34 (29.6)		8 (34.8)	26 (28.3)			
*KIR2DS4+*	*C2+*	204 (68)	178 (69.3)	0.784	44 (65.7)	134 (70.5)	0.773	0.616	0.538
	*C2–*	96 (32)	79 (30.7)		23 (34.3)	56 (29.5)			
	*C1+*	254 (84.7)	196 (76.3)	0.013 ^b^	51 (76.1)	145 (76.3)	0.105	0.024 ^d^	1.000
	*C1–*	46 (15.3)	61 (23.7)		16 (23.9)	45 (23.7)			
*KIR2DS5+*	*C1+*	70 (84.3)	70 (72.9)	0.072	9 (64.3)	61 (74.4)	0.129	0.127	0.517
	*C1–*	13 (15.7)	26 (27.1)		5 (100)	21 (25.6)			
	*C2+*	56 (67.5)	71 (74)	0.410	9 (64.3)	62 (75.6)	1.000	0.301	0.510
	*C2−*	27 (32.5)	25 (26)		5 (35.7)	20 (24.4)			

N is the total number of individuals; n is the number of individuals with the presence or absence of ligand for a *KIR* gene. Two-tailed Fisher’s exact test made comparisons. * The viruses analyzed were CMV, VHB, and VHC. P1, *p* value obtained by comparing total AC patients with healthy controls; P2 and P3, *p* value obtained by comparing AC patients with and without viral infection with healthy controls, respectively; P4, *p* value obtained by comparing AC patients without viral infection with AC with viral infection. ^a^, OR = 1.801; 95% IC: 1.155–2.809, *p* = 0.010; ^b^, OR = 1.719; 95% IC: 1.123–2.630, *p* = 0.013; ^c^, OR = 1.758; 95% IC: 1.092–2.831, *p* = 0.026; ^d^, OR = 1.714; 95% CI: 1.083–2.711, *p* = 0.024.

**Table 3:** Frequency of KIR genes and HLA-C genotypes in male AC patients with and without associated viruses compared with healthy controls.

			Male AC Patients						
		Healthy Control N = 319	Total Patients N = 281		with Virus N = 68	without Virus N = 213			
*KIR* Genes	*HLA-C* Genotypes	n (%)	n (%)	P1	n (%)	n (%)	P2	P3	P4
*iKIRs*									
*KIR2DL1+*/*S1−*	*C1C1*	62 (31.8)	47 (30.1)	0.817	15 (33.3)	32 (28.8)	0.860	0.609	0.570
	*C1C2*	104 (53.3)	76 (48.7)	0.393	22 (48.9)	54 (48.6)	0.622	0.476	1.000
	*C2C2*	29 (14.9)	33 (21.2)	0.159	8 (17.8)	25 (22.5)	0.648	0.118	0.666
*KIR2DL2+*	*C1C1*	62 (31.2)	43 (30.1)	0.905	17 (45.9)	26 (24.5)	0.090	0.236	0.021 ^a^
	*C1C2*	104 (52.3)	66 (46.2)	0.275	14 (37.8)	52 (49.1)	0.151	0.631	0.257
	*C2C2*	33 (16.6)	34 (23.8)	0.128	6 (16.2)	28 (26.4)	1.000	0.050	0.265
*KIR2DL3+*	*C1C1*	87 (31.8)	72 (29.9)	0.702	18 (30.5)	54 (29.7)	1.000	0.680	1.000
	*C1C2*	146 (53.3)	111 (46.1)	0.112	26 (44.1)	85 (46.7)	0.251	0.181	0.765
	*C2C2*	41 (15.0)	58 (24.1)	0.010 ^b^	15 (25.4)	43 (23.6)	0.057	0.026 ^c^	0.861
*aKIRs*									
*KIR2DS1+*	*C1C1*	38 (32.8)	34 (29.6)	0.786	8 (34.8)	26 (28.3)	1.000	0.546	0.611
	*C1C2*	59 (50.9)	50 (43.5)	0.293	7 (30.4)	43 (46.7)	0.109	0.579	0.239
	*C2C2*	19 (16.4)	31 (27.0)	0.056	8 (34.8)	23 (25.0)	0.079	0.164	0.431
*KIR2DS4+*	*C1C1*	96 (32.0)	79 (30.7)	0.784	23 (34.3)	56 (29.5)	0.773	0.616	0.538
	*C1C2*	158 (52.7)	117 (45.5)	0.106	28 (41.8)	89 (46.8)	0.137	0.228	0.568
	*C2C2*	46 (15.3)	61 (23.7)	0.013 ^d^	16 (23.9)	45 (23.7)	0.105	0.024 ^e^	1.000
*KIR2DS5+*	*C1C1*	27 (32.5)	25 (26.0)	0.410	5 (35.7)	20 (24.4)	1.000	0.301	0.510
	*C1C2*	43 (51.8)	45 (46.9)	0.551	4 (28.6)	41 (50.0)	0.150	0.877	0.159
	*C2C2*	13 (15.7)	26 (27.1)	0.072	5 (35.7)	21 (25.6)	0.129	0.127	0.517

N is the total number of individuals; n is the number of individuals with the presence or absence of the *KIR* gene. Two-tailed Fisher’s exact test made comparisons. Each *p* value was obtained by comparing each ligand with the other ligands for that specific gene. P1, *p* value obtained by comparing total male AC patients with healthy controls; P2 and P3, *p* value obtained by comparing male AC patients with and without viral infection and healthy controls, respectively; P4, *p* value obtained by comparing male AC patients with and without viral infection. ^a^ OR = 0.382; 95% IC: 0.175–0.837, *p =* 0.021; ^b^ OR = 0.555; 95% IC: 0.356–0.866, *p* = 0.010; ^c^ OR = 0.569; 95% IC: 0.353–0.916, *p* = 0.026; ^d^ OR = 0.582; 95% IC: 0.380–0.891, *p* = 0.013; ^e^ OR = 0.584; 95% IC: 0.369–0.923, *p =* 0.024.

## Data Availability

Data is unavailable due to privacy or ethical restrictions.
